# A Systematic Review on the Safety and Efficacy of PCSK9 Inhibitors in Lowering Cardiovascular Risks in Patients With Chronic Kidney Disease

**DOI:** 10.7759/cureus.29140

**Published:** 2022-09-13

**Authors:** Emmanuelar O Igweonu-Nwakile, Safina Ali, Salomi Paul, Shreyas Yakkali, Sneha Teresa Selvin, Sonu Thomas, Viktoriya Bikeyeva, Ahmed Abdullah, Aleksandra Radivojevic, Anas A Abu Jad, Anvesh Ravanavena, Chetna Ravindra, Prachi Balani

**Affiliations:** 1 Internal Medicine, California Institute of Behavioral Neurosciences & Psychology, Fairfield, USA; 2 Medicine, California Institute of Behavioral Neurosciences & Psychology, Fairfield, USA; 3 Behavioral Neurosciences and Psychology, California Institute of Behavioral Neurosciences & Psychology, Fairfield, USA; 4 General Surgery, California Institute of Behavioral Neurosciences & Psychology, Fairfield, USA

**Keywords:** evolocumab, dyslipidemia, alirocumab, safety of pcsk9 inhibitors, efficacy of pcsk9 inhibitors, hyperlipidemia treatment, risk factors cardiovascular diseases, chronic kidney disease (ckd), pcsk9 inhibitors

## Abstract

Cardiovascular events caused by dyslipidemia are one of the leading causes of death in patients with Chronic Kidney Disease (CKD). Statins are the first line of treatment for patients with hyperlipidemia and in the treatment regimen for patients with CKD. Therefore, the introduction of Proprotein Convertase Subtilisin-Kexin type 9 inhibitors (PCSK9 inhibitors) is a viable and possibly effective treatment option for patients who, despite high doses of statins, struggle to lower their low-density lipoprotein cholesterol (LDL-C) levels. Our paper's objective is to explore the safety of these biological agents, particularly in patients with varying stages of impaired kidney function, and the correlating effectiveness in lowering their LDL-C levels, thereby reducing cardiovascular risks in these patients.

We methodically retrieved relevant articles from PubMed, PubMed Central, Medline, and Google scholar in April 2022. We used the Medical Subject Heading (MeSH) Strategy and used the relevant keyword, then applied our inclusion and exclusion criteria; the initial search gave 10,542 results; with the removal of duplicates, irrelevant articles, and application of quality assessments done, we finally included 15 papers for our review with 37,188 patients.

PCSK9 inhibitors are reliable, safe, and efficient therapy in lowering LDL-C levels in patients with CKD. However, its safety and efficacy in severe and end-stage kidney disease are grey, as other factors such as infections lead to morbidity and mortality. Future trials on chronic kidney disease and PCSK9 inhibitors should investigate the inhibitors' ability to improve kidney functions at all stages of kidney disease while lowering lipid levels and finally analyze the safety in patients with end-stage kidney disease.

## Introduction and background

Approximately one in seven adults in the United States has Chronic Kidney disease (CKD) [1]. The numbers are even more astonishing when looking at the global burden; approximately 13.4% of the global population is recorded to have some form of CKD [2]. CKD is defined as renal damage or glomerular filtration rate of less than 60ml/min/1.72m^2^ for more than three months, irrespective of the cause [3].

Cardiovascular disease (CVD) is the most common cause of morbidity and mortality in patients with CKD. It is estimated that more than half of the deaths in CKD patients occur due to cardiovascular disease [4]. Among the traditional risk factors, hyperlipidemia has been associated with a worse cardiovascular prognosis in patients with CKD [4]. Changes in lipoprotein breakdown are common in CKD patients and may be linked to an increased rate of atherosclerosis and CVD [5]. The lipoprotein profile in the early stages of CKD is characterized by hypertriglyceridemia and low high-density lipoprotein (HDL) cholesterol but normal low-density lipoprotein cholesterol (LDL-C) levels. Altogether, this combination of lipid abnormalities contributes to the elevated incidence of premature CVD in CKD patients. Lowering LDL cholesterol (LDL-C) is beneficial for preventing major atherosclerotic events in these patients [5,6]. Given the high risk of cardiovascular disease, lipid-lowering agents have been widely accepted in preventing cardiovascular outcomes in CKD patients. Statins have been the most commonly used and taken drugs. Still, clinicians face some challenges, including the inability of some patients to achieve optimal lipid levels of < 70mg/dl at a maximum dose of statins [7], some patients still experience cardiovascular events while on the maximally tolerated dose of statins and a small number of patients are intolerant to statins [8].

The introduction of Proprotein Convertase Subtilisin-Kexin type 9 inhibitors (PCKS9 inhibitors) have shown to reduce low-density lipoprotein cholesterol (LDL-C) by about 45-55% when added to background statin, and this reduces cardiovascular events [7]. PCSK9 is a secreted serine protease that binds to the extracellular domain of the hepatocyte low-density lipoprotein receptor (LDL-receptor) and leads to its lysosomal destruction [7]. Loss-of-function mutations in *PCSK9* were determined to reduce LDL-C levels by 15-28% and the risk of coronary heart disease by 47-88%, so monoclonal antibodies that act as PCSK9 inhibitors sequester PCSK9 and thereby prevent LDL receptor destruction, leading to an increase in LDL receptor density on hepatocytes and lowering of LDL-C levels [6].

This systemic review investigates if PCSK9 inhibitors are safe for patients at all stages of CKD and if they prevent cardiovascular events when combined with statins or used alone in statin-intolerant patients.

## Review

Methods

While writing this paper, we adhered to the Preferred Reporting Items for Systematic Reviews and Meta-Analysis (PRISMA) guidelines [9]. We researched literature for relevant data collection by searching multiple electronic databases, including PubMed, Medline, PubMed Central (PMC), and Google Scholar. We utilized the terms for Medical Subject Heading (MeSH) strategy using the keywords arranged into the following concepts: Concept A, “Chronic Kidney Disease"; Concept B, “Hyperlipidemia” OR “DYSLIPIDEMIA”; Concept C; “Proprotein Convertase Subtilisin/Kexin Type 9 Inhibitors (PCSK9 Inhibitors )” OR “Alirocumab” OR “Evolocumab”; Concept D: "Hydroxymethylglutaryl-Coa Reductase Inhibitors” OR “Statins” OR“ Atorvastatin “OR “ Simvastatin.” We searched these concepts in two combinations. We had an initial search result of 1,912 from PubMed, PubMed Central, And Medline and an initial search result of 8,360 from Google Scholar. Table 1 below depicts our search strategy for electronic databases. 

**Table 1:** Search Strategy for Electronic Database PCSK9 inhibitors: Proprotein Convertase Subtilisin/Kexin Type 9 (PCSK9) inhibitor

Search Strategy	Database	Number of articles	After Inclusion and Exclusion Criteria Were Applied
B+C Hyperlipidemias/drug therapy"[Majr] AND "PCSK9 Inhibitors/adverse effects"[Majr] OR “Alirocumab” OR “Evolocumab”	PUBMED, PubMed Central, MEDLINE	1262	113
A+B+C+D ( "Renal Insufficiency, Chronic/complications"[Majr] OR "Renal Insufficiency, Chronic/drug therapy"[Majr] OR "Renal Insufficiency, Chronic/mortality"[Majr] OR "Renal Insufficiency, Chronic/prevention and control"[Majr] )AND "Hyperlipidemias/drug therapy"[Majr] AND "PCSK9 Inhibitors/adverse effects"[Majr] OR “Alirocumab” OR “Evolocumab” AND ( "Hydroxymethylglutaryl-CoA Reductase Inhibitors/adverse effects"[Majr] OR "Hydroxymethylglutaryl-CoA Reductase Inhibitors/poisoning"[Majr] OR "Hydroxymethylglutaryl-CoA Reductase Inhibitors/toxicity"[Majr] ) OR ( "Atorvastatin/adverse effects"[Majr] OR "Atorvastatin/poisoning"[Majr] OR "Atorvastatin/toxicity"[Majr] ) OR ( "Simvastatin/adverse effects"[Majr] OR "Simvastatin/poisoning"[Majr] OR "Simvastatin/toxicity"[Majr] )	PUBMED, PubMed Central, PUBMED,MEDLINE	650	14
Chronic kidney disease AND Dyslipidemia AND PCSK9 Inhibitors	Google Scholar	8,630	5,550

Inclusion Criteria 

We included articles written in English and published within the last five years. We had studies conducted on adult humans who were 19 years and older. The studies included randomized control trials, clinical trials, case controls, cross-sectional and cohort studies, and traditional and systematic reviews.

Exclusion Criteria 

We excluded grey literature, animal studies, studies not in English, Books, and literature.

Results 

The initial search through the electronic databases identified 10,542 studies; after applying the inclusion and exclusion criteria, 5,677 studies remained. After careful automated and manual removal of duplicates, 2,100 studies remained. A further selection of relevant articles based on appropriate titles and relevant abstracts left 54 studies for full-text review. A 50-70% benchmark was established for the articles to be assessed for quality assessment, and they all qualified. Finally, we have included 15 studies in the review.

**Figure 1:**
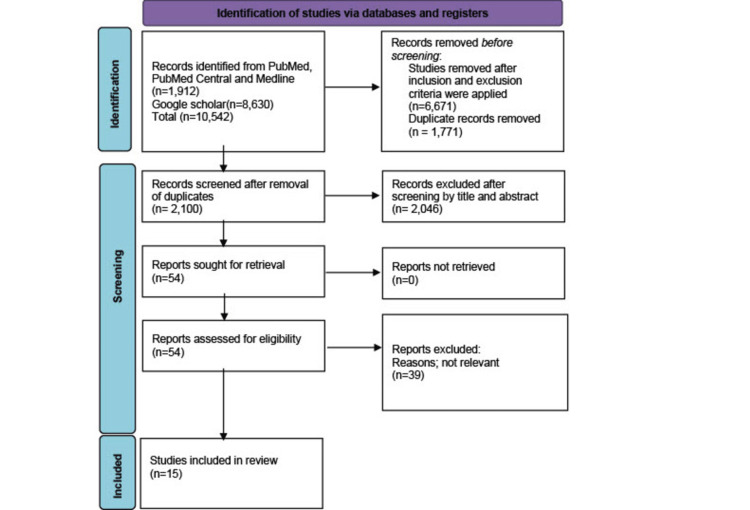
PRISMA (Preferred Reporting Items for Systematic reviews and Meta-Analysis) Flowchart Presenting Study Selection Strategy

The quality assessment tools used include; the Newcastle-Ottawa scale for observational studies (cohort studies and case-control), AXIS-appraisal tool for cross-sectional studies, AMSTAR-appraisal tool for systematic reviews and meta-analysis, and SANRA-scale for the assessment of narrative review articles. Our systematic review includes 15 studies, 10 systematic reviews, three randomized control trials, and two pooled randomized control trials with 37,188 patients. Figure 1 below shows the PRISMA flow chart [9].

Discussion

We conducted a systematic review on PCSK9 inhibitors’ safety in patients at all stages of kidney impairment. Also, we assessed its ability to lower cardiovascular events when combined with statins or used alone in statin-intolerant patients.

The Pathophysiology of Dyslipidemia in Patients with Chronic Kidney Disease (CKD)

Dyslipidemia is common in patients with CKD and increases as the stage progresses. The causes are changes in the lipoprotein structure and reverse cholesterol transport, modified metabolism of triglycerides, and increased levels of lipoprotein(a) [4].

Low-density lipoprotein (LDL) cholesterol and total cholesterol are usually within the standard value range in patients with CKD [6]. However, with an alteration in the structure and a predominance of small dense low-density lipoprotein (LDL) over other LDL sub-fractions, this small dense LDL has an increased capacity to infiltrate the arterial intima [6]. In healthy patients, reverse cholesterol transport removes excess cholesterol from the arterial wall, but in patients with CKD, this is significantly reduced [6]. This is due to the decreased activity of the adenosine tri-phosphate binding cassette transporters (ABCA1, ABCR1) in promoting the affluence of cholesterol from macrophages to lipid-poor high-density lipoprotein (HDL) precursors [6]. Additionally, the maturation of HDL precursors from free cholesterol by the plasma enzyme lecithin cholesterol acyltransferase (LCAT) is reduced due to the low levels of LCAT activating apolipoprotein A1 (APOA1) [6]. As a result, cholesterol esters are transferred from HDL particles to triglyceride-rich lipoprotein mediated by cholesteryl ester transfer protein (CETP). These impaired metabolisms lead to the accumulation of immature HDL, increasing the risk of atheroma formation [4].

There is an increased plasma concentration of triglycerides in patients living with CKD [6]. The increased level of apolipoprotein C III (APO CIII) in patients with CKD inhibits the activity of lipoprotein lipase in hydrolyzing triglycerides transported within very low-density lipoprotein (VLDL) particles and chylomicrons [6]. As a result, the expression of VLDL receptors in adipocytes and myocytes in patients with CKD is reduced; this prevents VLDL clearance from circulation and its transformation into intermediate-density lipoprotein (IDL) particles. In addition, glucose loading and recurrent heparinization in patients on peritoneal dialysis and hemodialysis reduce these receptors [6]. Finally, lipoprotein(a) inhibits fibrinolysis and promotes atherosclerosis. Patients with CKD have elevated lipoprotein(a) levels due to decreased clearance [6].

The Mechanism of Action of PCSK9 Inhibitors

The scientific community’s interest was piqued when discovering a mutation in the PCSK9 gene led to the understanding of autosomal dominant hypercholesterolemia (ADH) [10]. Furthermore, in 2003, a paper published showed how a gain of function mutation in PCSK9 in French families led to very high levels of LDL-C and ultimately familial hypercholesterolemia [10,11].

**Figure 2:**
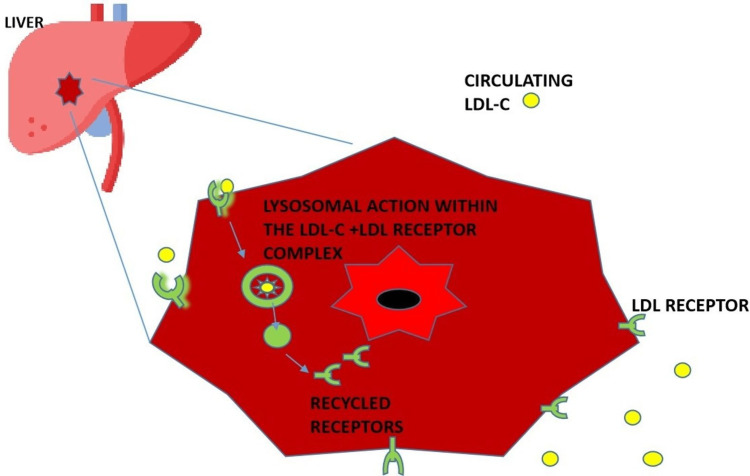
Diagram showing the normal degradation of LDL-C in the hepatocyte and recycling of LDL receptor to hepatocyte surface LDL: Low-density lipoproteins, LDL-C: Low-density lipoproteins-cholesterol. "liver Icon made by Freepik from www.flaticon.com" The main illustration was created by the first author, Emmanuelar O. Igweonu-Nwakile.

PCSK9 is a protein produced in the liver, kidney, and small intestine. It plays a significant role in the metabolism of low-density lipoprotein cholesterol (LDL-C) and LDL receptors [11,12]. LDL receptors in the hepatocytes remove circulating LDL-C by engulfing it in an endosome, where lysosomes degrade it. The LDL receptors then dissociate and are recycled back to the surface of the hepatocyte, where they continue to bind to circulating LDL-C [10-13]. However, increased levels of PCSK bind to LDL receptors, such that when LDL-C binds to a receptor that has an attached PCSK9 molecule, it leads to the lysosomal breakdown of both the LDL-C and the LDL receptors; hence no recycling of the receptors back to the hepatic surface, and this eventually leads to excessive amounts of LDL-C in the circulation [10-13]. Figure 2 illustrates the expected degradation of LDL-C in the liver and the recycling of LDL receptors to the surface of the hepatocyte.

**Figure 3:**
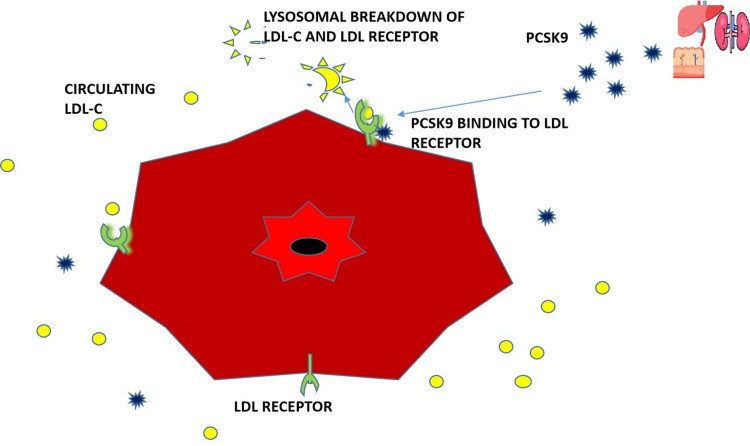
Diagram showing PCSK9 bound to LDC-c +LDL receptor promoting the degradation of both the receptor and LDL-c. LDL: Low-density lipoprotein, LDL-C: Low-density lipoprotein-cholesterol, PCSK9: Proprotein Convertase Subtilisin-Kexin type 9 "Liver icon created by Freepik from www.flaticon.com" "Kidney icon created by Krismaker from www.flaticon.com" "Small intestine icon created by Freepik from www.flaticon.com" The main illustration was created by the first author, Emmanuelar O. Igweonu-Nwakile.

**Figure 4:**
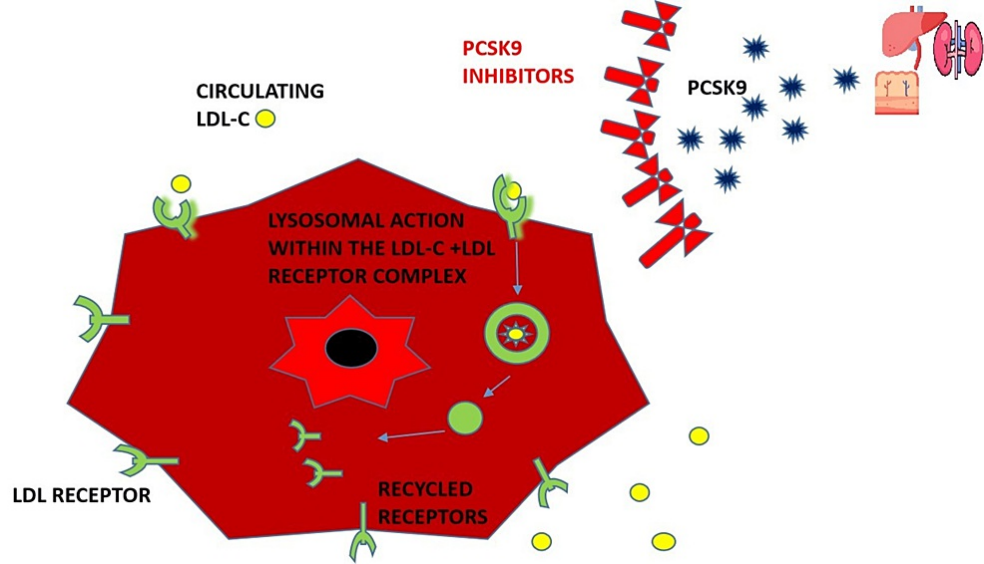
Diagram showing the introduction of PCSK9 inhibitors which prevent the degradation of LDL receptors, allowing the recycling of the receptor. LDL: Low-density lipoprotein, LDL-C: Low-density lipoprotein-cholesterol PCSK9 inhibitors; Proprotein Convertase Subtilisin-Kexin type 9 inhibitors "Liver icon created by Freepik from www.flaticon.com" "Kidney icon created by Krismaker from www.flaticon.com" "Small intestine icon created by Freepik from www.flaticon.com" The main illustration was created by the first author Emmanuelar O. Igweonu-Nwakile.

Figure 3 illustrates the effect of PCSK9 on the LDL-C and LDL receptor complex, which leads to the breakdown of both, thereby preventing the recycling of LDL receptors and Figure 4 illustrates the introduction of PCSK9 inhibitors; this prevents the binding of PCSK9 to the LDL-C and LDL receptor complex, thereby allowing for the recycling of LDL receptors back to the surface of hepatocytes and therefore lowering LDL-C levels in the body. 

Evolocumab and alirocumab are the current PCSK9 inhibitors in the market today; they were released in 2015 as add-on therapy to statins when LDL-C levels rise above 150mg/dl or 135mg/dl in patients at increased cardiovascular risk [12,13]. The third inhibitor made, bococizumab, is a humanized Monoclonal Antibody (Mab) with about 3% of the murine sequence in its antigen-binding complementarity-determining region; this could have led to the severe immunogenic reactions and injection site reactions that were reported, unlike the other two drugs, which were fully human monoclonal antibodies [8].

PCSK9 Inhibitors and Chronic Kidney Disease: Safety, Adherence, and Efficacy of Lowering Cardiovascular Risks

The largest published clinical trial on PCSK9 inhibitors in CKD patients was the FOURIER trial by Charytan et al. in 2019, which had 27,565 participants who were administered evolocumab 140 mg bi-weekly or 420 mg monthly; they were randomly given evolocumab or a placebo in a 1:1 ratio while all being on background statins [7]. The study showed a 59% reduction in LDL-C levels in patients on evolocumab regardless of kidney function [7]. In other words, evolocumab showed an identical ability to lower LDL-C levels in patients with normal kidney function and those with stage two and three CKD [7]. Similarly, the relative risk reduction for the primary endpoint and key secondary cardiovascular outcomes was identical in patients across all stages of CKD [7]. The trial pointed out that the drug's benefits in preventing cardiovascular events became less effective as the estimated glomerular filtration rate (eGFR) declined; patients were presumed to die from non-atherosclerotic causes such as low immunity leading to infections and arrhythmias [7].

The pooled analysis of eight randomized trials by Toth et al. also had similar findings to the FOURIER trial; their study of alirocumab in CKD had 4,629 participants with hypercholesterolemia, 10% of whom had CKD [14]. They were all on background statins; at week 24, the LDL-C levels had reduced by 46.1% to 62.1%, averaging 54.1% [14]. There was a similar improvement across the entire lipid profile of participants [14]. Noticeably, the renal function in participants of the pooled analysis did not change; there were no improvements or declinations in renal function [14]. Both the FOURIER trial in 2019 and the pooled analysis in 2018 did not include participants with eGFR of less than 20ml/min/1.73m^2^ and less than 30ml/min/1.73m^2^, respectively, so the ability to ascertain the safety of patients in this population cannot be proven [7,14]. Furthermore, we cannot confirm if these drugs have a role in improving renal function [7,14].

The Moriarty et al. open-label trial on the efficacy and safety of alirocumab in statin-intolerant patients over three years showed a 45% reduction in LDL-C levels when used alone, compared to a 14.6% reduction when ezetimibe was used in 2019 [15]. The study shows the ability to improve cardiovascular events when used alone to manage dyslipidemia [15].

One of the factors that contribute to the efficacy of PCSK9 inhibitors is that, as an injectable biologic drug, it is given once bi-weekly or once monthly; the pooled randomized study by Farnier et al. investigated the one to two-year adherence of patients taking alirocumab showed a 98% adherence rate compared to 80% in patients taking oral agents such as statins [16]. Treatment adverse effects that led to discontinuation include myalgia and injection-site soreness, leading to a 1% discontinuation rate [16]. However, there was a consistent rate of LDL-C reduction at an average of 53.8% [16].

A leading cause of CKD is long-standing diabetes, which leads to diabetic nephropathy; the 2017 randomized clinical trial by Leiter et al. showed that patients with Type 1 and Type 2 Diabetes Mellitus (DM) who were on insulin benefitted from the introduction of alirocumab in addition to statins or alirocumab as a monotherapy in patients who are statin intolerant [17]. At week 24, there was a 47.8% reduction in LDL-C in patients with Type 1 DM and a 49% reduction in patients with Type 2 DM [17]. Since statins have been implicated in causing insulin resistance in some patients, this study has shown that replacing them with a PCSK9 inhibitor will achieve similar or sometimes better results in reducing LDL-C, thereby preventing cardiovascular events [17].

In the bid to lower the cardiovascular risk in patients with dyslipidemia, thinking the lower the LDL-C level, the better, some concerns have been raised from a physiological standpoint on the concept of "too low LDL-C"; these include the development of cataracts, de novo diabetes, and most importantly, the development of hemorrhagic strokes [18]. However, the review by Quiroga et al. in 2020 carefully debunked all these concerns based on a lack of evidence and confirmed that the levels of LDL-C that PCSK9 inhibitors can achieve are safe [18]. Furthermore, an American woman who was found to have inherited a loss of function mutation in *PCSK9* from both parents led to no PCSK9 activity, and her lifetime LDL-C level was at an average of 14mg/dl [13]. She went on to live a healthy life with none of the concerning side effects listed above [13]. Finally, the most benefit in lowering cardiovascular events are seen in patients with the most risk and who are administered the inhibitors the longest [19]. Table 2 below depicts the characteristics of the studies that describe the safety and efficacy of PCSK9 inhibitors in patients with normal, threatened, and impaired kidney functions.

**Table 2:** Table depicting the characteristics of papers which compared the safety and efficacy of PCSK9 inhibitors in patients with preserved, threatened and impaired kidney functions LDL: Low-density lipoprotein, LDL-C: Low-density lipoprotein-cholesterol, PCSK9: Proprotein Convertase Subtilisin-Kexin type 9, CKD: Chronic kidney disease

Study	Author	Year	Type of Study	Number of Patients	Purpose of Study	Results	Conclusion
1	Quiroga et al. [18]	2020	Review	NR-not reported	To investigate the safety of Proprotein Convertase Subtilisin-Kexin type 9 (PCSK9) inhibitors in chronic kidney disease (CKD) and assess the safety of low levels of Low-density lipoprotein cholesterol (LDL-C) levels from a physiological standpoint	PCSK9 inhibitors are safe in mild and moderate stages of CKD, and there were no proven effects of low levels of LDL-C.	PCSK9 inhibitors are safe to use in chronic kidney disease
2.	Charytan et al. [7]	2019	Randomized clinical trial	27,564	To investigate the outcomes of patients with varying degrees of kidney function when taking Evolocumab versus a placebo	A total of 27,564 patients participated in the study. There was a 59% reduction in LDL-C with Evolocumab compared to patients taking the placebo.	When given in addition to statins in patients with impaired kidney functions, Evolocumab led to improved LDL-C levels. However, patients with end-stage kidney disease were not included in the study, so the lack of adverse effects cannot be entirely ascertained.
3	Moriarty et al. [15]	2019	Odyssey open-label clinical trial trial	281	The purpose of the study was to investigate the long-term safety and efficacy of Alirocumab in patients with moderate to high levels of cardiovascular risks who are intolerant to statins	There was a reduction of 45% in LDL-C patients taking Alirocumab vs 14.6% in those taking ezetimibe as an alternative	A population of statin-intolerant patients was used and it was found that pcsk9 inhibitors are efficacious in lowering LDL-C levels and reducing cardiovascular risks
4	Wong et al. [19]	2019	Review	NR- Not reported	To review multiple trials on the efficacy and safety of PCSK9 inhibitors in lowering cardiovascular events	Pcsk9 inhibitor improved the entire lipid profile, and decreased cardiovascular events such as myocardial infarction and strokes	PCSK9 inhibitors were safe and efficacious
5	Toth et al. [14]	2018	Pooled randomized clinical trials	4629	The aim was to ascertain the safety of Alirocumab in patients with CKD, considering that pcsk9 is expressed in the kidneys	A total of 4629 participants with hypercholesteremia from eight pooled randomized phase three odyssey trials participated and 10% of the participants had different degrees of renal impairment, there was an average of 54% reduction in LDL-C across all patients	Although there was a reduction in LDL-C levels, there was no improvement in kidney functions. The safety of the biology in patients with end-stage renal disease could not be ascertained
6	Farnier et al. [16]	2017	Pooled randomized clinical trial	4197	To investigate the one-two year adherence rate of patients taking 75mg to 150mg of Alirocumab bi-weekly, the patient population being those with sub-optimally controlled hypercholesteremia. It also aims to investigate the efficacy of lowering LDL levels over one-two years	There was a 98% adherence rate in patients taking Alirocumab is 98% with minimum side effects such as myalgias and injection site soreness. There was an LDL-C reduction of 53.8%	patients who are more likely to be adherent to bi-weekly or monthly injectables than daily oral medications
7.	Leiter et al. [17]	2017	Randomized clinical trial	517	To investigate the safety and efficacy of alirocumab in preventing cardiovascular events in patients with type one or Type two diabetes who were on insulin	By week 24, there was a 47.8% reduction in LDL-C levels in patients with type one Diabetes Mellitus and a 49% reduction in patients with type two Diabetes mellitus	Diabetic nephropathy is the leading cause of chronic kidney disease in patients; Alirocumab has been shown to reduce the LDL-C levels in these patients and those who are statin resistant reducing their cardiovascular risks

Limitations

Several studies included were systematic and traditional reviews, so the risk for observational bias is present. We excluded some relevant studies written in Spanish due to the language barrier. There were no trials that included participants with severe and end-stage kidney disease. There are no long-term trials (following participants for >10 years) to ascertain the long-term safety of PCSK9 inhibitors or to ascertain the improvement of kidney functions in these patients.

## Conclusions

The efficacy of PCSK9 inhibitors in lowering LDL-C levels is consistent in all studies in patients with both preserved and impaired kidney functions. In addition, the safety of PCSK9 inhibitors in patients with mild and moderate CKD is consistent in all the studies reviewed. Still, we cannot ascertain the safety in patients with eGFR of less than 20ml/min/1.73m^2^ since trials did not include this population.

PCSK9 inhibitors work well alone and when combined with moderate to high-strength statins; in other words, PCSK9 inhibitors can be used in statin-intolerant patients. There is a clear relationship between PCSK9 inhibitors’ ability to improve lipid profile and lower cardiovascular risks, leading to lower morbidity and mortality rates. However, that relationship becomes skewed at severe stages of CKD, where non-thrombotic causes lead to morbidity and mortality. More trials are needed to ascertain long-term safety and improvement in CKD patients with PCSK9 inhibitors. Future research should study the safety and efficacy of PCSK9 in patients with CKD three and higher. We currently do not have enough knowledge of PCSK9 inhibitors and the different modes of dialysis, like hemodialysis versus peritoneal dialysis. Finally, the role of PCSK9 inhibitors in renal transplant patients would also be an exciting study in the future. 
